# Transcriptome landscape of *Synechococcus elongatus* PCC 7942 for nitrogen starvation responses using RNA-seq

**DOI:** 10.1038/srep30584

**Published:** 2016-08-04

**Authors:** Sun Young Choi, Byeonghyeok Park, In-Geol Choi, Sang Jun Sim, Sun-Mi Lee, Youngsoon Um, Han Min Woo

**Affiliations:** 1 Clean Energy Research Center, Korea Institute of Science and Technology, Hwarang-ro 14-gil 5, Seongbuk-gu, Seoul 02792, Republic of Korea; 2 Green School (Graduate School of Energy and Environment), Korea University, 145 Anam-ro, Seongbuk-gu, Seoul 02841, Republic of Korea; 3 Department of Biotechnology, Korea University, 145 Anam-ro, Seongbuk-gu, Seoul 02841, Republic of Korea; 4 Department of Chemical and Biological Engineering, Korea University, 145 Anam-ro, Seongbuk-gu, Seoul 02841, Republic of Korea; 5 Department of Clean Energy and Chemical Engineering, Korea University of Science and Technology, 217 Gajeong-ro, Yuseong-gu, Daejeon 34113, Republic of Korea

## Abstract

The development of high-throughput technology using RNA-seq has allowed understanding of cellular mechanisms and regulations of bacterial transcription. In addition, transcriptome analysis with RNA-seq has been used to accelerate strain improvement through systems metabolic engineering. *Synechococcus elongatus* PCC 7942, a photosynthetic bacterium, has remarkable potential for biochemical and biofuel production due to photoautotrophic cell growth and direct CO_2_ conversion. Here, we performed a transcriptome analysis of *S. elongatus* PCC 7942 using RNA-seq to understand the changes of cellular metabolism and regulation for nitrogen starvation responses. As a result, differentially expressed genes (DEGs) were identified and functionally categorized. With mapping onto metabolic pathways, we probed transcriptional perturbation and regulation of carbon and nitrogen metabolisms relating to nitrogen starvation responses. Experimental evidence such as chlorophyll *a* and phycobilisome content and the measurement of CO_2_ uptake rate validated the transcriptome analysis. The analysis suggests that *S. elongatus* PCC 7942 reacts to nitrogen starvation by not only rearranging the cellular transport capacity involved in carbon and nitrogen assimilation pathways but also by reducing protein synthesis and photosynthesis activities.

Global concerns about energy security and environmental issues affecting climate changes have focused attention on engineering photosynthetic organisms that are able to sequester and convert CO_2_ to organic materials using solar energy^1^,^2^. In order to improve the strains, system-wide engineering and optimization is required to reduce the time-, cost-, and labor-intensive process of strain development^3^. In particular, systems biology including omics analysis has been integrated to strain development.

Nitrogen is an essential element of all the complex macromolecules in a bacterial cell. Anthropogenic nitrogen supply in the presence of CO_2_ is one of the critical factors in the occurrence of cyanobacterial blooms mainly caused by *Microcystis* spp.^4^. On the other hand, a nitrogen limitation strategy is commonly used to maximize production of the desired product such as triacylglycerol content in microalgae in the carbon partitioning mechanisms^5^ and to trigger polyhdroxyalkanoate (PHA) accumulation as carbon storage in *Ralstonia eutropha*^6^ and in engineered *Escherichia coli*^7^. In addition, improvement of isoprenoid production (astaxanthinin *Haematococcus pluvialis*^8^,^9^ and amorpha-4,11-diene in *E. coli*^10^) has been reported under nitrogen limitation. Thus, it is important to understand the positive and negative mechanisms of nitrogen regulation in a cell for system-wide strain development.

Systems biology-based analyses have revealed the cellular mechanisms for nitrogen regulation in industrially-relevant bacteria. Nitrogen assimilation and amino acid biosynthesis of *Corynebacterium glutamicum*, an industrial amino acid producer, have been studied using a DNA microarray^11^. Chromatin immunoprecipitation followed by high-throughput sequencing (ChIP-seq) has been applied to address the links between the nitrogen stress response and stringent response in *E. coli* by identifying genome-wide DNA binding sites for the global transcriptional regulator NtcC that controls gene expression for nitrogen responses^12^. In addition, metabolic and transcriptomic responses of a wine yeast (*Saccharomyces cerevisiae* strain EC1118) have been investigated under a nitrogen-limited condition, showing its responses related to wine quality^13^.

The nitrogen starvation response of cyanobacteria including *Synechococcus elongatus* PCC 7942 as a cyanobacterial model organism has been studied to understand the mechanisms of degradation of phycobiliproteins^14^ and its consequent induction of chlorosis^15^,^16^. A low level of photosynthesis has been shown for long-term adaptation to nitrogen starvation^15^. Increased expression of NblA (a small polypeptide) was found to be responsible for degradation of phycobiliproteins using mutant screening under a nitrogen deprivation condition^17^, which is regulated by the global regulator NtcA for nitrogen control^18^,^19^. In addition, the signal transduction protein P_II_ and the regulator PipX (P_II_ interacting protein X) also modulate the gene expression in nitrogen control in *S. elongatus* PCC 7942^20^,^21^. The proposed regulatory model of PipX showed that NtcA-independent regulons are involved in the adaption of cyanobacteria for translation and photosynthesis to nutritional changes. However, two-dimensional proteomic analysis has failed to identify proteins for comprehensive nitrogen regulation due to the low amount of protein synthesized during nitrogen starvation^16^. Recently, transcriptomic and phosphoproteomic analyses of *Synechocystis sp.* PCC 6803 for nitrogen starvation have been reported using DNA microarray^22^ and LC-MS/MS^23^, respectively. However, a global transcriptomic analysis of *S. elongatus* PCC 7942 has not been available yet for the nitrogen starvation response although a comprehensive analysis of *S. elongatus* PCC 7942 using ChIP-seq, RNA-seq, and tiling expression microarray has been reported^24^.

Here, our study aims to understand the transcriptional landscape of *S. elongatus* PCC 7942 for the nitrogen starvation response using RNA-seq. Differentially expressed genes were identified with functional categories and nitrogen starvation-related metabolic metabolisms. The transcriptomic patterns and regulation obtained from the RNA-seq analysis were supported with additional experimental evidence. The results provided insight into the nitrogen starvation responses in *S. elongatus* PCC 7942 for system-wide metabolic engineering.

## Results

### Nitrogen starvation response of *S. elongatus* PCC 7942

**Figure 1:**
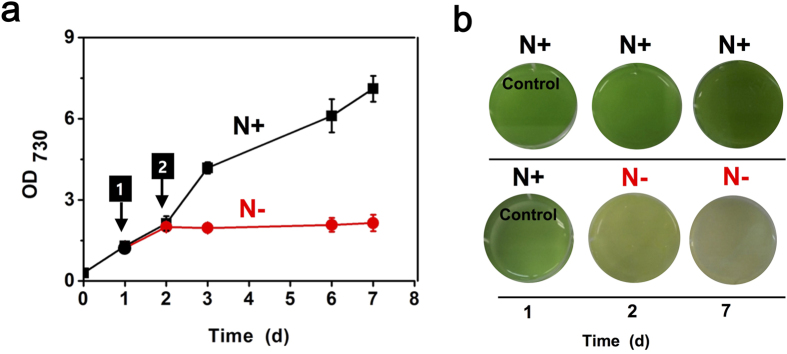
Growth of *S. elongatus* PCC 7942 for nitrogen starvation response. (**a**) Growth of the wild type grown in either BG-11 medium (N+; black square) or nitrogen-free BG-11 medium (N**−**; red circle) was measured at OD_730_. Arrow 1 and 2 indicated the time points of sampling for RNA-Seq: from duplicate cultures. (**b**) Images of cyanobacterial cell suspension of either a control (N+) or a nitrogen-starved cell (N**−**) were shown in 1 d, 2 d, and 7 d.

In order to investigate the nitrogen starvation responses of *S. elongatus* PCC 7942, cyanobacterial cells were cultivated in either BG-11 medium (N**+**) or nitrogen-free BG-11 medium (N**−**). Cyanobacterial cell growth was stopped under a N**−** condition only, resulting in chlorosis of cells after days of starvation in the medium (Fig. 1), which has been shown previously^25^. Subsequently, we analyzed the transcriptome of *S. elongatus* PCC 7942 for nitrogen starvation responses using RNA-seq. The biological duplicated samples were collected after 24 h of the nitrogen starvation.

### Global transcriptomic analysis of *S. elongatus* PCC 7942 for nitrogen starvation response

**Figure 2:**
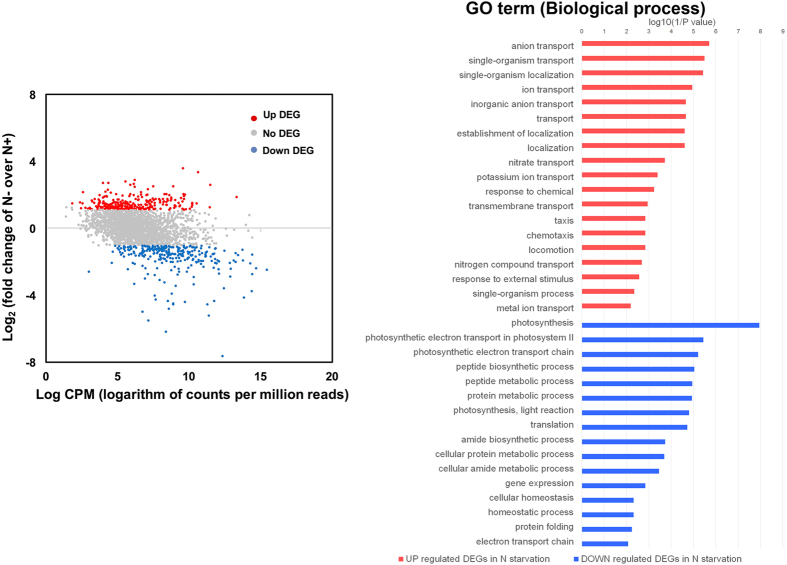
Statistical analysis and GO enrichment analysis of differential gene expression and categories of differentially expressed genes in *S. elongatus* PCC 7942 for nitrogen starvation (N−) vs. nitrogen repletion (N+). Plots of log_2_ ratio (fold change of nitrogen starvation (N**−**) vs. nitrogen repletion (N+)) vs. the number of the log counts per million reads in the two conditions for nitrogen starvation (N**−**) vs. nitrogen repletion (N+) in *S. elongatus* PCC 7942. Red or blue dots indicate genes detected as differentially expressed with p-value ≤ 0.01 and either higher than 1 or less than -1, respectively. Differentially expressed genes (p-value ≤ 0.01 and log_2_ |fold change| ≥1) were categorized by clusters of orthologous groups of proteins (COG) based on the biological process.

Compared to cells grown under a nitrogen repletion condition, differentially expressed genes (DEGs) of *S. elongatus* PCC 7942 under a nitrogen starvation condition were identified by the R bioconductor RNA-seq analysis pipeline and filtered with criteria of both p-value ≤ 0.01 and log_2_ |fold change| (log|FC|) ≥1. Among 545 differentially expressed genes, 284 genes were down-regulated and 261 genes were up-regulated (Fig. 2 and Supplementary Table S1). The highly up-regulated genes (logFC ≥3) for nitrogen starvation responses were mostly annotated as hypothetical proteins having no COGs (Clusters of Orthogonal Groups). On the other hand, the most down-regulated genes (logFC ≤-6) for nitrogen starvation responses were a gene encoding for rubrerythrin (a non-heme iron protein) that has been related to oxidative stress protection^26^ and a gene encoding a hypothetical protein.

In order to find functional categories of DEGs, we performed a GO enrichment analysis over biological processes, molecular functions, and cellular locations. As shown in Fig. 2, many cellular transporter activities were up-regulated for nitrogen starvation responses, i.e. ion, nitrate, and nitrogen compound transport, whereas photosynthesis and protein translation process were down-regulated.

### Transcriptomic analysis in specific metabolisms of *S. elongatus* PCC 7942 for nitrogen starvation response

**Figure 3:**
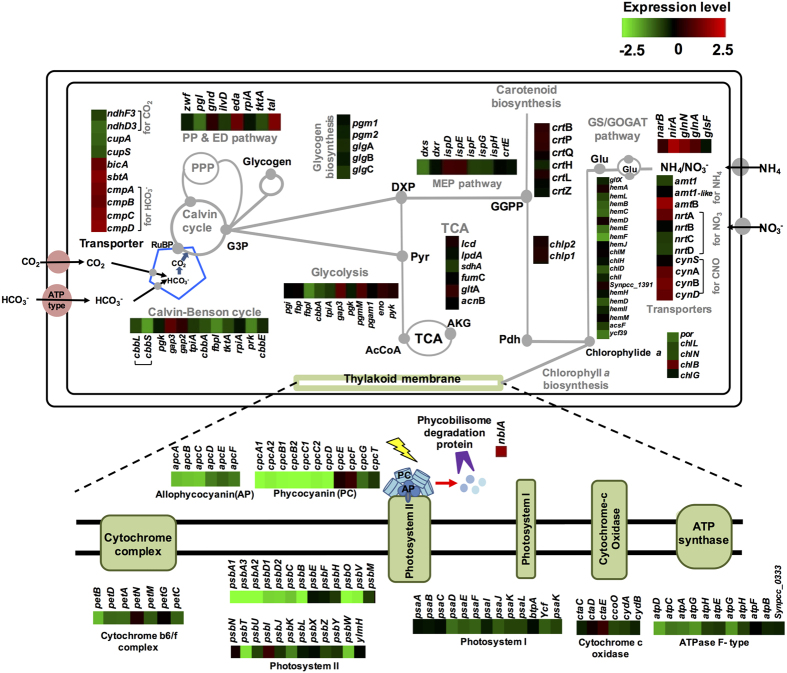
Cellular metabolism comparing carbon and nitrogen assimilations and photosynthesis with the differentially expressed genes in *S. elongatus* PCC 7942 for nitrogen starvation (N−) vs. nitrogen repletion (N+). Differentially expressed genes (p-value ≤ 0.01 and log_2_ |fold change| ≥1) were shown in different metabolic pathways using heat maps with gene names or accession IDs. Metabolic pathways of *S. elongatus* PCC 7942 include carbon assimilation and nitrogen assimilation (GS/GOGAT pathway), transporter systems, and secondary metabolic pathways (methylerythritol phosphate pathway, MEP; chlorophyll *a* biosynthesis; carotenoid biosynthesis). Abbreviations: Pentose phosphate pathway, PPP; glyceraldehyde-3-phosphate, GAP; glucose-3-phosphate, G3P; pyruvate, Pyr; 1-deoxy-D-xylulose 5-phosphate, DXP; α-ketoglutarate, AKG (or 2-OG); Acetyl-CoA, AcCoA; geranylgeranyl diphosphate, GGPP; phytyl diphosphate, Pdh; L-glutamate, Glu; L-glutamine, Gln. The photosynthesis overall component contains photosystem I and photosystem II with APC and PC, cytochrome *b*_*6*_*f* complex and cytochrome *c* oxidase, and ATP synthase in thylakoid membrane.

Stringent responses of nitrogen starvation have been shown in the cellular transporters to adapt environmental changes^12^. Here we described the transcriptional outlook of specific metabolisms over nitrogen starvation responses in *S. elongatus* PCC 7942 with RNA-seq analysis (Fig. 3 and Supplementary Table S1).

#### Nitrogen assimilation

For ammonium uptake, three cyanobacterial ammonium transporters (Amt1, AmtB, and Amt1-like) were annotated in *S. elongatus* PCC 7942. The expression of gene encoding NtcA-regulated AmtB (encoded by locus *Synpcc7942_2279*) is responsible for nitrogen limitations, which was up-regulated by 1.7 of logFC after 24 h of nitrogen starvation. On the other hand, transcriptional levels of genes encoding for Amt1 (encoded by locus *Synpcc7942_0442*) and Amt1-like (encoded by locus *Synpcc7942_0965*) were not differently altered. Besides ammonium, nitrate and urea are commonly used by cyanobacteria as a nitrogen source. An ABC-type transporter constituted by NrtABCD (encoded by locus *Synpcc7942_1239-1236*), which is involved in nitrate-nitrite uptake^27^, were up-regulated (1.1 of logFC). Also, a NtcA-regulated ferredoxin-nitrite reductase NirA (encoded by locus *Synpcc7942_1240*) was up-regulated (1.9 of logFC) to the nitrogen starvation response. However, a ferredoxin-nitrate reductase NarB (encoded by locus *Synpcc7942_1235*) was not up-regulated. Genes encoding for urease and urea transporters meanwhile have not identified in *S. elongatus* PCC 7942 and their orthologous genes predicted by the orthologs of *Synechocystis* sp. PCC 6803 were not differently expressed upon nitrogen starvation. Interestingly, an ABC-type cyanate transporter and a cyanate lyase (encoded by the *cynDBAS* genes; *Synpcc7942_2107-2104*) were up-regulated (1.1 of logFC). Thus, nitrogen-starved cyanobacterial cells transport cyanate and irreversibly catalyze bicarbonate-dependent conversion of cyanate to ammonium^28^.

Further assimilation of ammonia into glutamine and glutamate, the GS/GOGAT pathway was used to subsequently distribute nitrogen to other cellular components in *S. elongatus* PCC 7942. Expressions of the *glnN (Synpcc7942_0169*) gene encoding for glutamine synthase (Type III GS) were differentially up-regulated by 1.5 of logFC due to transcriptional activation by NtcA. However, expressions of the *glnA (Synpcc7942_2156*) gene encoding for the type I GS and the *glsF (Synpcc7942_0890*) gene encoding for a ferredoxin-dependent glutamate synthase (GOGAT; Glutamine oxoglutarate aminotransferase) were not differentially up-regulated by 0.9 and 0.4 of logFC, respectively.

Nitrogen control is regulated by a transcriptional regulator NtcA (encoded by locus *Synpcc7942_0965*). As a RNA-seq result, NtcA was positively auto-regulated (1.4 of logFC) to the nitrogen starvation response and regulated transcriptional expressions of the *amtB, nirA, glnN*, and *cynDBAS* genes by activating NtcA-promoters. Another regulon of NtcA is the *glnA (Synpcc7942_2156*) and *glnB (Synpcc7942_0321*) genes encoding for glutamine synthase (GS) and P_II_ protein, respectively^27^. However, RNA-seq showed that the *amt1, glnA*, and *glnB* genes were not transcriptionally activated after 24 h of nitrogen starvation. Gene expression of NtcB (LysR family of transcriptional regulator), encoded by locus *Synpcc7942_1242*) and PipX (encoded by *Synpcc7942_2061*), a co-activator of NtcA, was not differentially changed.

#### Carbon assimilation

Compared with bicarbonate transporters of *Synechocystis* sp. Strain PCC 6803^29^, single bicarbonate component BicA (encoded by locus *Synpcc7942_1380*), a constitutive high-flux low-affinity Na^+^/HCO_3_^−^ symport, SbtA (encoded by locus *Synpcc7942_1475*), an inducible low-flux high-affinity Na^+^/HCO_3_^−^ symport were differently expressed (logFC ≥1) in a nitrogen starvation condition. Also, a multicomponent ATP-binding bicarbonate transporter BCT1 (encoded by the *cmpABCD* genes; *Synpcc7942_1488-1491*) was differently expressed (logFC ≥1) in a nitrogen starvation condition. SbtA and CmpABCD were up-regulated regardless of inorganic carbon conditions. Besides bicarbonate transporters, the multiple NADH:ubiquinone oxidoreductase complex is essential to the CO_2_ uptake system in cyanobacteria^30^. In *S. elongatus* PCC 7942, a small NADH:ubiquinone oxidoreductase (NDH-1S) encoded by the *ndhF3*-*ndhD3*-*cupAS (Synpcc7942_2091-2094*) gene clusters plays a CO_2_ uptake system, which were not down-regulated in nitrogen starvation (N**−**) compared with nitrogen (N+), although the genes were down-regulated in nitrogen starvation (N**−**) compared with a nitrogen starvation control (N**−**_Ctr_) (see the details in Supplementary Table S1). Furthermore, genes encoding for NDH-1L and NDH-1M were not altered.

#### Carbon assimilation pathway and central metabolisms

Nitrogen starvation in *S. elongatus PCC 7942* did not alter significant expression of genes encoding for β-carboxysome^31^, which acts as a microcompartment to supply CO_2_ to Rubisco along with carboxysomal CA activity. No particular changes were shown for expression of carbonic anhydrase encoded by *ccaA (Synpcc7942_1447*), *ecaA (Synpcc7942_1388*), and *ccmM (Synpcc7942_1423*). The carbon fixing enzyme of the Calvin-Basham-Benson (CBB) cycle, Rubisco encoded by the *cbbLS (Synpcc7942_1426-1427*) genes, is crucial for carbon assimilation in cyanobacteria. Expression of the *cbbLS* genes was not differentially expressed for the nitrogen starvation. Unlike CbbLS, expression of the *prk (Synpcc7942_0977*) encoding for phosphoribulokinase and *fbpI (Synpcc7942_0505*) encoding for the type I fructose-1,6-bisphosphatase in the CBB pathway was down-regulated in both nitrogen starvation (N**−**) compared with nitrogen (N+) and nitrogen starvation (N**−**) compared with a nitrogen starvation control (N**−**_Ctr_) by logFC ≤−1. However, expressions of the remaining genes in the CBB pathway were not differentially changed.

In the glycolytic pathway, the pentose phosphate pathway, and the TCA cycle, no differentially expressed genes were found except genes encoding enzymes that involved a reaction with G3P as either a substrate or a product such as Gap3 (encoded by locus *Synpcc7942_1939*), Eda (encoded by locus *Synpcc7942_0017*), or Tal (encoded by locus *Synpcc7942_2297*). Furthermore, expressions of genes in glycogen biosynthesis were not changed.

#### Secondary metabolic pathways

*S. elongatus* PCC 7942 possesses the methylerythritol phosphate (MEP) pathway, which utilizes glyceraldehyde-3-phosphate and pyruvate to produce IPP and DMAPP for terpenoids biosynthesis (i.e. geranylgernaly diphosphate; GGPP). The terpenoid pathway in cyanobacteria is linked to chlorophyll *a* biosynthesis^32^. Geranylgernaly reductase converts GGPP to phytyl diphosphate (Pdh), which is used as a substrate for chlorophyll synthase (ChlG encoded by locus *Synpcc7942_2084*) to produce a chlorophyll *a* for photosynthesis. Another co-substrate for ChlG is a chlorophyllide *a*, which is produced from L-glutamate via both chlorophyllide *a* biosynthesis and tetrapyrrole biosynthesis.

As a result of RNA-seq for nitrogen starvation response, expressions of most genes involved in the MEP pathway were not changed except the *dxs (Synpcc7942_0430*) encoding for deoxyxyulose-5-phosphate synthase, which is the first enzyme of the MEP pathway. Dxs was down-regulated (logFC ≤−1) in *S. elongatus* PCC 7942 for the nitrogen starvation condition. On the other hand, genes in involved in chlorophyllide *a* and tetrapyrrole biosynthesis were significantly changed by logFC ≤−1.

#### Photosynthetic and respiratory electron transport pathways

Cyanobacteria utilize redox-active components in thylakoids for both photosynthesis and respiration^33^. For oxygenic photosynthesis, both photosystem I and II are required. In photosystem II (PSII), allophycocyanin (APC) and phycocyanin (PC) are a light-harvesting pigment-protein complex of the phycobiliproteins of cyanobacteria. Type-I NADPH dehydrogenase oxides NADPH and reduces plastoquinone (PQ) with PS II. Electrons are transported from the PQ pool to the cytochrome *b*_*6*_*f* complex. Soluble electron carriers such as plastocyanin or cytochrome *c* reduce the oxidized photosystem I (PSI) and a single electron is transferred to NADP. Reduced NADP can be used for CO_2_ fixation. A proton gradient from the electron transport pathway is used for ATP synthesis.

RNA-seq for the nitrogen starvation response revealed that genes encoding for PSII and its phycobilisome in *S. elongatus* PCC 7942 were differentially expressed by logFC ≤−4.2. Also, several genes encoding for F_0_F_1_ATPase were altered by logFC ≤−1. Genes encoding for NDH-1L and NDH-1M complexes, cytochrome *b*_*6*_*f* complex, PS I, and cytochrome *c* oxidase were not significantly altered under nitrogen starvation. Interestingly, NblA, a phycobilisome degradation protein encoded by the *nblA (Synpcc7942_0430*), was significantly up-regulated by 1.96 of logFC, resulting in chlorosis in *S. elongatus* PCC 7942 under nitrogen starvation.

### Biological evidences for down-regulated photosynthesis and up-regulated bicarbonate transporter of *S. elongatus* PCC 7942 for nitrogen starvation response

Based on the transcriptomics analysis, two distinct features were shown for nitrogen starvation responses in *S. elongatus* PCC 7942. First, genes encoding for APC and PC and its biosynthesis pathway (chlorophyll *a* biosynthesis) were significantly down-regulated. On the other hand, genes encoding for bicarbonate uptake transporters were up-regulated for nitrogen starvation. Thus, we performed biological experiments to support the findings from the RNA-seq analysis.

**Figure 4:**
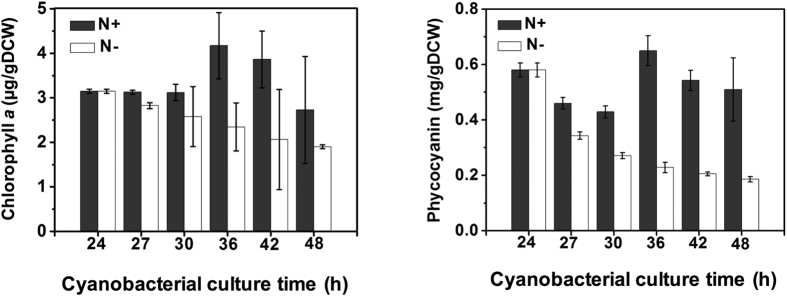
Decreased chlorophyll *a* and phycocyanin contents of *S. elongatus* PCC 7942 under nitrogen starvation condition. The levels of chlorophyll *a* and phycocyanin contents (mg/gDCW) were measured by the previous spectrophotometric assay^14^ under either nitrogen starvation (N**−**; white bar) or nitrogen repletion (N+; black bar) condition. Note that ‘24 h’ and ‘48 h’ on the time axis corresponds to the time point of the arrow 1 and arrow 2 of the Fig. 1 as the cyanobacterial culture time (h), respectively. Data are presented as the mean of at least three independent experiments. The error bars represent the standard deviation of samples.

**Figure 5:**
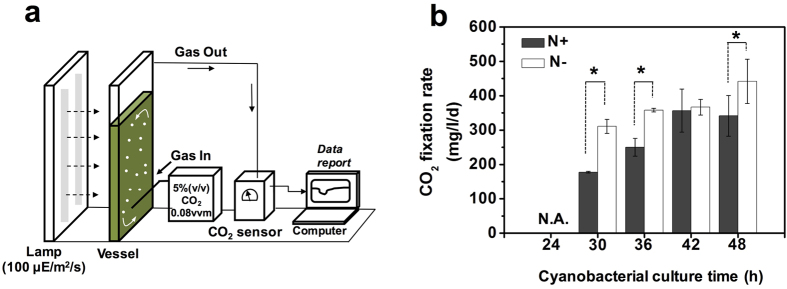
Alleviated initial CO_2_ fixation rate of *S. elongatus* PCC 7942 under nitrogen starvation condition. (**a**) a cartoon of the controlled photobioreactor setting for cultivation of *S. elongatus* PCC 7942 in 1.8 L BG-11 medium either with or without nitrogen sources, supplied with bubbled air containing 5% CO_2_ at a flow rate of at flow rate of 140 mL min^−1^ under constant illumination light (100 μmol photons m^−2^ s^−1^). The concentration of CO_2_ was measured using an infrared by CO_2_ analyzer (Q-S153, Qubit systems, Kingston, ON, Canada) with a digital data recording computer. (**b**) Measurement of CO_2_ fixation rates of *S. elongatus* PCC 7942 under either nitrogen starvation (N**−**; white bar) or nitrogen repletion (N+; black bar) condition in a controlled photobioreactor. Note that ‘24 h’ and ‘48 h’ on the time axis corresponds to the time point of the arrow 1 and arrow 2 of the Fig. 1 as the cyanobacterial culture time (h), respectively. The calculation of CO_2_ fixation rate (mg/l/d) was described in the section of method. Data are presented as the mean of at least duplicate independent experiments. The error bars represent the standard deviation of samples (*p-value < 0.01).

To confirm whether the levels of Chlorophyll *a* (Chl *a*) and phycobiliproteins (APC and PC) were decreased under nitrogen starvation, we determined the levels of Chl *a* and PC content in *S. elongatus* PCC 7942 using a spectrophotometer. As a result, both levels of Chl *a* and PC content of *S. elongatus* PCC 7942 were gradually decreased after nitrogen starvation stress was induced (Fig. 4), consistent with previous results^25^. Eventually, chlorosis of the cyanobacterial cells occurred. Subsequently, we investigated whether the CO_2_ fixation rate was changed in *S. elongatus* PCC 7942 under a nitrogen starvation condition. Thus, we performed the measurement in a controlled photobioreactor supplied with bubbled air containing 5% CO_2_ (Fig. 5). The results showed that the CO_2_ fixation rates for the nitrogen starvation condition were statistically higher than for the nitrogen repletion condition (p-value < 0.01), which corresponds to the experimental conditions for the transcriptomics. However, the difference in the CO_2_ fixation rates diminished over the cell culture.

## Discussion

Although there are many questions with respect to the regulation of nitrogen control, there is evidence that regulation of the expression of genes involved in nitrogen assimilation in cyanobacteria involves interactions of P_II_-protein X (PipX) with the global regulator NtcA and the specific DNA binding of NtcA to target sites^20^,^21^. Consistently, the Ntc target genes such as the *ntcA, amtB, nirA, glnN*, and *cynDBAS* genes were up-regulated for nitrogen starvation conditions in our results. However, some target genes listed^12^ were not significantly regulated. The reason remains unclear, although they conserved the DNA binding motif of NtcA. Our RNA-seq analysis did not reveal that the nitrogen starvation response is regulated by PipX alone in an NtcA-independent manner. Thus, a high resolution ChIP-seq analysis for NtcA or PipX could be useful to provide accurate information on transcriptional regulation. Nonetheless, up-regulation of an AmtB transporter, ferredoxin-nitrite reductase, and Type II glutamine synthase (GlnN) in the nitrate assimilation system must be critical for *S. elongatus* PCC 7942 to respond to nitrogen starvation for 24 h.

Moreover, regulation of NtcA is also involved in ammonium assimilation into carbon metabolism^27^. Nitrogen starvation is perceived as an increase in the concentration of intracellular 2-oxoglutrate, which acts as a signal molecule for intracellular sensor P_II_ protein. Accumulation of the levels of 2-oxoglutarate (2-OG) modulates the phosphorylation status of P_II_ protein^34^,^35^ to interact with PipX for further transcriptional regulation with NtcA. Besides serving as a substrate for the GS/GOGAT pathway, 2-OG is a product or substrate in the 2-OG decarboxylating TCA cycle. When expressions of metabolic enzymes that consume or produce 2-OG were investigated, expressions of genes encoding for isocitrate dehydrogenase (*Synpcc7942_1719*) and 2-OG decarboxylase (*Synpcc7942_1435*) were not changed. Thus, increased levels of 2-OG must be due to high expression of the GS/GOGAT activity in nitrogen starved *S. elongatus* PCC 7942, which was also confirmed by our RNA-seq analysis. Moreover, NtcA regulates OmpR-type regulator Rre37, which is involved in nitrogen-expression of sugar catabolic genes in *Synechocystis* sp. 6803^36^. Homology of Rre37 in *S. elongatus* PCC 7942 is annotated in a transcriptional regulator but it has not been characterized yet.

Furthermore, protein P_II_ co-regulates inorganic carbon uptake in cyanobacteria^37^,^38^. Given that phosphorylated P_II_ is related to high CO_2_ concentration (5% CO_2_) and low nitrogen availability, the mutant *ΔglnB* (encoding for P_II_ of *Synechocystis* sp. 6803) showed high affinity to high CO_2_ concentration. In addition, decreased phycocyanin content and increased glycogen content have been shown in the mutant *ΔglnB*^39^. However, the effect is limited on the metabolome and the inorganic carbon uptake in *S. elongatus* PCC 7942^40^. Similarly, expression of the *glnB* genes in this study was not significantly changed for the nitrogen starvation condition. Thus, the role of P_II_ signaling protein is important to decipher carbon and nitrogen metabolisms with the status of phosphorylation, which could be useful for metabolic engineering of cyanobacteria.

The global regulatory model for carbon and nitrogen assimilation involves the levels of 2-OG in expression of the high affinity carbon concentrating mechanism. It has been reported that CcmR of *Synechocystis* sp. 6803 negatively regulates expression of genes for SbtA and NDH-1S transporters in the presence of co-repressors (NADP+ and 2-OG)^41^. Interestingly, our RNA-seq study showed that gene expressions for carbon uptake transport (BicA, SbtA, and BCT1) were up-regulated for the nitrogen starvation condition in *S. elongatus* PCC 7942. CmpR of *S. elongatus* PCC 7942 has been shown to be a transcriptional activator for the *cmp* operon encoding for a BCT1 transporter in the presence of 2-phosphoglycolate as a co-activator^42^. Up-regulation of SbtA under nitrogen starvation remains unclear because CcmR could be missing in *S. elongatus* PCC 7942. These up-regulations of bicarbonate transporters under nitrogen starvation led us to investigate the CO_2_ uptake rate, and thereupon a significant increase of the CO_2_ uptake rate was measured under a nitrogen starvation condition in a controlled photobioreactor (Fig. 5). Interestingly, genes of a distinct cyanate transporter of *S. elongatus* PCC 7942 were highly up-regulated, which might also be involved in an increase of the CO_2_ uptake rate by catalyzing a bicarbonate-dependent conversion of cyanate to supply ammonium. Thus, an increased CO_2_ uptake rate could be an initial nitrogen starvation response.

The stringent response in photosynthesis is regulated with C and N metabolism in *S. elongatus* PCC 7942 under a nitrogen starvation condition. Previously, accumulation of intracellular glycogen has been observed in nitrogen-starved cyanobacteria, in contrast with decreased levels of total lipid and total protein contents. However, our experiment did not show the DEGs for glycogen biosynthesis. However, genes coding for Gap3, Eda, and Tal (G3P consuming or producing enzymes) were highly up-regulated, which might be related to accumulation of glycogen under nitrogen starvation. Thus, a comprehensive gene expression analysis could be required to understand the genetic regulation of accumulation of glycogen under nitrogen starvation responses in cyanobacteria. We speculated that a signal protein P_II_-PipX complex could be related to the genetic regulation of biosynthesis of glycogen under the nitrogen limitation stress responses in cyanobacteria, which is currently being investigated. Our study also confirmed that expression of the *nblA* gene was positively regulated by NtcA under nitrogen starvation. Phycobilisome as 30% of total proteins in a cell^18^ can be degraded to supply an alternative nitrogen source by the increased activity of NblA. Its degradation process has been shown to be related to acclimation and long-term survival for cyanobacterial cells^14^,^16^. In addition, a decrease of the first enzyme (Dxs) of the MEP pathway under nitrogen starvation is related to shutting down biosynthetic metabolism for photosynthesis as a stringent response.

## Conclusion

A transcriptome analysis of *S. elongatus* PCC 7942 under a nitrogen starvation condition was provided in the point of the global regulatory network of carbon and nitrogen assimilation. Genes involved in carbon and nitrogen assimilation were highly up-regulated. Photosynthesis and its related metabolic genes were significantly down-regulated. Detailed information of RNA-seq for nitrogen starvation in *S. elongatus* PCC 7942 could be useful for metabolic engineering to accelerate the development of biosolar cell factories to re-direct carbon flux of CO_2_ to desired products under a nitrogen limiting condition.

## Methods

### Cyanobacterial strain and growth conditions

*S. elongatus* PCC 7942 strain was grown at 30 °C while shaking under constant illumination light (100 μmol photons m^−2^ s^−1^) in BG-11 medium as described previously^2^. For nitrogen starvation experiments, cyanobacterial cells were grown in 50 ml BG-11 for 24 h as a control (N**+**) and the cells at OD_730_ of 1.0 were harvested by centrifugation at 13 000 rpm for 5 min. Then, cells pellets were re-suspended either in fresh BG-11 medium (N**+**) or nitrogen-free BG-11 medium (N**−**) after washing twice (refer ‘arrow 1’ in Fig. 1a). The cells were cultivated for 24 h and the biological duplicated samples were prepared for RNA-seq (refer ‘arrow 2’ in Fig. 1a) and stored at −80 °C until they were analyzed.

### RNA preparation and RNA-seq

5 mL of Trizol reagent (California, Carlsbad, USA) was added to the cyanobacterial cell pellet from 50 mL culture under liquid nitrogen. To break the cell wall, the quick frozen cell was grinded using a mortar and pestle and aliquot into 1 mL tube. After addition 200 μl of 1-bromo-3-chloropropane in a 1 mL tube and incubation for 3 min at room temperature, the extraction mixture was centrifuged at 13 000 rpm for 15 min 4 °C. Retrieved aqueous phase was mixed with the equal volume of isopropanol and incubated for 10 min at room temperature and centrifuged to concentrate the precipitated RNA at 4 °C. The RNA pellet was washed using 75% (v/v) ethanol (1 mL), air dried and finally dissolved in 30 μL RNase-free water. The quantity and quality of total RNA were evaluated using RNA electropherograms (Agilent 2100 Bioanalyzer, Agilent Technologies, Santa Clara, USA). Total RNA (10 μg) from each sample was used as a starting material to prepare sequencing libraries. The Ribo‐Zero rRNA removal kit (Epicentre, USA) was used for ribosomal RNA depletion according to manufacturer instructions. Libraries for Illumina sequencing were made with the TruSeq Stranded mRNA sample prep kit (Illumina, USA) following the manufacturer’s protocol. RNA sequencing was performed on the Illumina HiSeq 2500 platform using single‐end 50 bp sequencing. All RNA‐sequencing and alignment procedures were conducted by ChunLab (Seoul, South Korea). All data sets have been uploaded to the Gene Expression Omnibus under accession (GSE79726) and all sequencing reads were submitted to NCBI Sequence Read Archive (SRP072154).

### Data analysis and Statistical analysis

The quality of raw reads was checked by Fast QC and the reads with low quality (Q <30) were eliminated using HTQC^17^. Bowtie2^17^ software was used to align the reads with the *S. elongatus* PCC 7942 reference genome. The relative transcript abundance was quantified as raw read counts and reads per kilobase per million mapped reads (RPKM). edgeR^43^, a Bioconductor components in R packages was used for differential gene expression analysis. Cyanobacterial gene ontology analysis was performed by assigning COG (Clusters of Orthologous Groups) categories with KEGG (Kyoto Encyclopedia of Genes and Genomes) database. In addition, cyanobacterial clusters of orthologous groups of proteins (CyoOG)^44^ categories to each gene of *S. elongatus* PCC 7942 were assigned (Supplementary Table S1). Heat maps generated by MeV (MutiExperiment Viewer ver. 4.8) showed differential expressed genes for nitrogen starvation responses in Fig. 3. For gene ontology enrichment analysis, a GO term annotation file obtained by IPR2GO (http://genome.microbedb.jp/CyanoBase) was used to perform an hypergeometic test with unsupported model organisms using the GOstat^45^ of R package. Then, a custom data structure of the GO ontologies was created.

### Measurement of Chlorophyll *a* and phycocyanin contents using spectrophotometer

For the measurement of Chlorpholly *a* (Chl *a*) and phycocyanin (PC) contents, cyanobacterial cell cultures (1 mL) were harvested at the cyanobacterial culture time and the pigments of cell pellets were extracted in 100% acetone at 50 °C for 15 min. After centrifugation (13 000 rpm for 3 min), the pigment extract was analyzed for Chl *a* (mg per mL) by following the previous spectrophotometric assay^14^.

The supernatant was obtained and the absorbance was measured at 620, 680, and 750 nm on Cary 60 UV-Vis spectrophotometer (Agilent technologies, CA, USA). For PC measurement, harvested cell suspension (1 mL) was treated either with or without heating at 75 °C for 8 min. PC was determined by measuring the absorbance at 620 nm and 750 nm and PC content (mg per mL) was calculated by following the previous spectrophotometric assay^14^.

### Calculations for carbon fixation rate using CO_2_ analysis

Cyanobacterial cells were inoculated to OD_730_ of 1 in 1.8 L of either BG-11 medium (N**+**) or nitrogen-free medium (N**−**) in a flat-panel photobioreactor (Labfors 5 Lux-LED flat-panel option [637 mm (L) × 298 mm (W) × 79 mm (D); INFORS-HT, Bottmingen, Switzerland]) under constant illumination light (100 μmol photons m^−2^ s^−1^). 5% CO_2_ (v/v) and 95% air (v/v) was supplied directly into the medium at flow rate of 140 mL min^−1^ (0.08 vvm) by gas bubbling and 5 N NaOH was used to adjust pH 7 in the medium. CO_2_ analysis was performed by continues monitoring CO_2_ concentration from off-gas line before and after cell inoculation. CO_2_ concentration (ppm) was digitally recorded every per second by CO_2_ analyzer (Q-S153, Qubit systems, Kingston, ON, Canada).

The carbon fixation rate in a unit of mg of CO_2_ per liter per day was calculated from the previous calculation^1^. Briefly, CO_2_ value was converted from ppm into μmol CO_2_ L^−1^ using an equation of 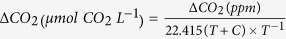, where *C* refers to the temperature in °C ant T refers to the absolute temperature (273K). Based on the flow rate (in L min^−1^) (i.e., 140 mL min^−1^) and the volume of the culture medium (i.e., 1.8 L), the CO_2_ fixation rate can be obtained with units of mg l^−1^ d^−1^.

## Additional Information

**How to cite this article**: Choi, S. Y. *et al*. Transcriptome landscape of *Synechococcus elongatus* PCC 7942 for nitrogen starvation responses using RNA-seq. *Sci. Rep.* **6**, 30584; doi: 10.1038/srep30584 (2016).

## Supplementary Material

Supplementary Information
